# POFUT1 as a cancer biomarker: insights into its oncogenic mechanisms and clinical relevance

**DOI:** 10.1007/s00109-025-02578-1

**Published:** 2025-08-07

**Authors:** Oumaima Mazour, Sébastien Legardinier, Bouabid Badaoui, Agnès Germot

**Affiliations:** 1 LABCiS (Laboratory of Agroresources, Biomolecules and Chemistry for Health of Innovation), University Limoges, UR 22722, F-87000 Limoges, France; 2 Laboratory of Biodiversity, Ecology and Genome, Department of Biology, Faculty of Sciences, University Mohammed V, B.P 1014 RP, 10100 Rabat, Morocco

**Keywords:** Cancer biomarker, *O*-fucosylation, Oncogene, POFUT1, Signaling pathway, Tumor microenvironment

## Abstract

Over the past decade, increasing evidence has linked the dysregulation of human protein *O*-fucosyltransferase 1 (POFUT1), overwhelmingly through gene overexpression, to tumor progression in multiple cancers, including colorectal, breast, gastric, lung, hepatocellular carcinoma, and glioblastoma. This review provides a comprehensive analysis of the molecular and cellular consequences of POFUT1 dysfunction in cancer. *POFUT1* overexpression driven by copy number variations (CNVs), epigenetic alterations, and/or post-transcriptional modifications enhances tumorigenesis by activating key oncogenic pathways such as Notch, Wingless-type MMTV integration site family (Wnt)/β-catenin, and phosphoinositide 3-kinase/protein kinase B/mammalian target of rapamycin (PI3K/AKT/mTOR). These pathways promote cell proliferation, migration, and epithelial-mesenchymal transition (EMT) while simultaneously suppressing apoptosis. Additionally, POFUT1 promotes an immunosuppressive tumor microenvironment that contributes to treatment resistance by immune checkpoint inhibitors. Mainly, its overexpression is detectable at early stages of tumor development and in some cancer patient sera, highlighting its potential as a non-invasive biomarker for early cancer detection and disease monitoring. Given its role in immune evasion and therapy resistance, POFUT1 represents a promising therapeutic target, warranting further investigation into its clinical applications.

## Introduction

**Fig. 1:**
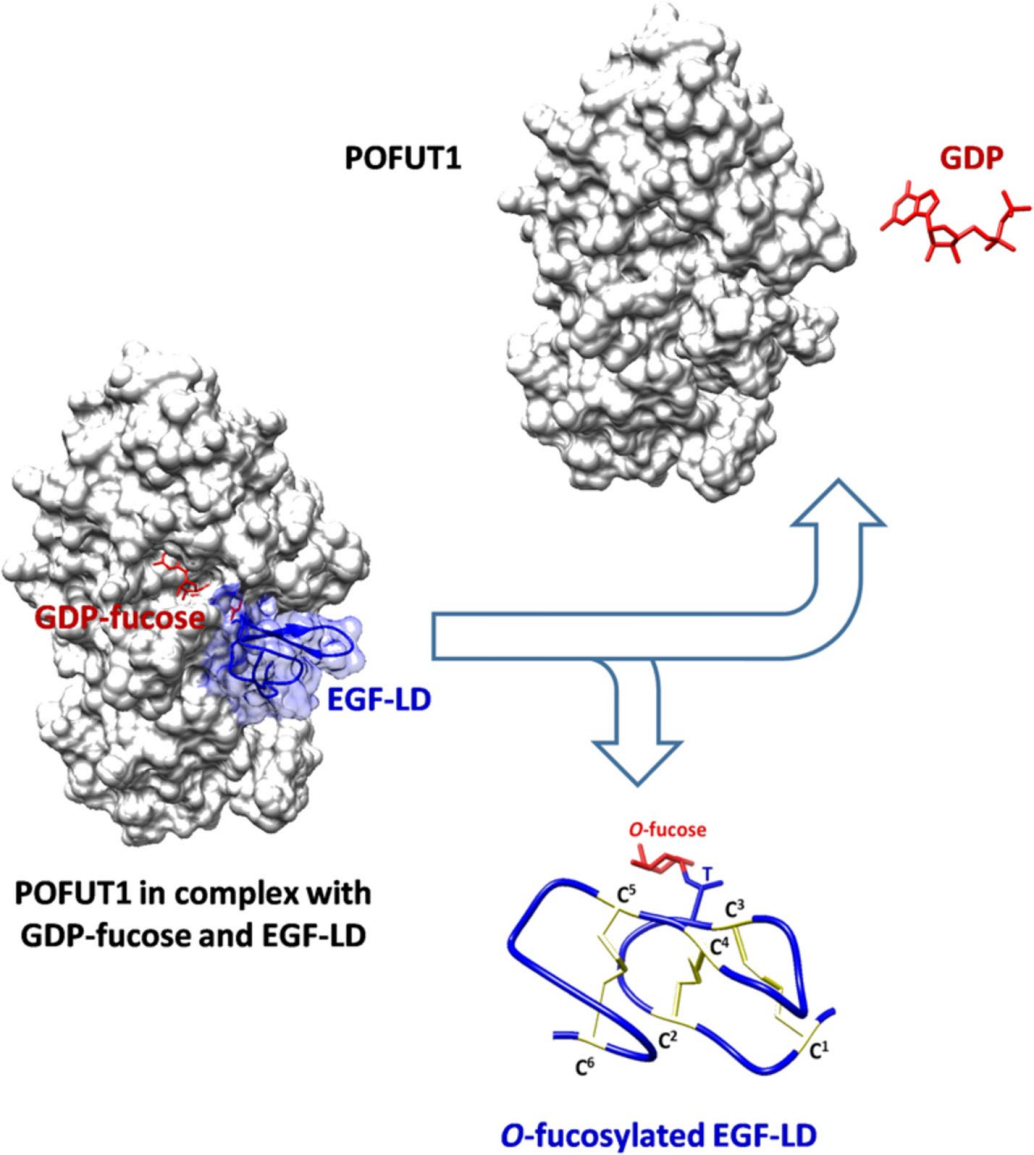
*O*-fucosylation of EGF-like domain by POFUT1. On the left, POFUT1 complexed with its donor substrate, namely the GDP-fucose, is shown interacting with an EGF-LD (mainly found in secreted and cell-surface membrane glycoproteins). By cleaving GDP-fucose, POFUT1 directly adds fucose via an *O*-linkage to threonine (T), in the example, of the *O*-fucosylation site (C^2^XXXXS/TC^3^) of the correctly folded EGF-LD. C^2^ and C^3^ are the second and third of the six conserved cysteine involved in three disulfide bridges stabilizing the protein domain

Human protein *O*-fucosyltransferase 1 (POFUT1) is an endoplasmic reticulum-localized glycosyltransferase encoded by a gene located on chromosome 20 [1, 2]. This polyexonic gene spans 30,779 base pairs and produces a protein-coding transcript of 5226 bases. *Pofut1* is a single copy gene in animal genomes and its exon–intron organization is conserved among vertebrates [3]. It encodes in humans a mature functional enzyme consisting of 362 amino acids, which belongs to the GlycosylTransferase family 65 [4]. This glycosyltransferase catalyzes the addition of a fucose from GDP-L-fucose on hydroxyl groups of serine (S) or threonine (T) residues within the consensus sequence C^2^XXXXS/TC^3^ present in epidermal growth factor-like domains (EGF-LDs) of glycoproteins (Fig. 1). Here, C^2^ and C^3^ represent the second and third cysteine residues [2, 5] among the six conserved ones usually found in EGF-LDs [6]. There are approximately 100 human protein targets of POFUT1, which possess at least one EGF-LD with a potential *O*-fucosylation site [7]. A number of these human proteins, such as AGRIN (involved in the development of the neuromuscular junction), VWA2 (an extracellular matrix protein with a high gene expression in colorectal cancer cells [8]), and uPA (urokinase-type plasminogen activator, used as a tumor prognostic marker), were shown to be *O*-fucosylated by POFUT1, which is essential for their correct folding, stability, and functional activity [7, 9, 10]. Among the other POFUT1 protein targets, NOTCH receptors have been the most studied so far due to the high numbers of EGF-LDs with *O*-fucose residues on their NECD (Notch extracellular domain) and their central role in the Notch signaling pathway involved in cell–cell communication [11, 12]. In mice, the knockout of *Pofut1* results in embryonic lethality at ≈ E10.5–E11.5 affecting somitogenesis, vasculogenesis, cardiogenesis, and neurogenesis, with phenotypes closely resembling those observed in the disruption of the NOTCH signaling pathway [13]. In *Pofut1*^Cax/Cax^ mice, which harbor a hypomorphic mutation in the *Pofut1* gene, the reduced expression of *Pofut1* has been associated with hypertrophied muscle fibers and a decline in satellite cell populations, implicating distortion in the balance of proliferative/differentiated cells [14, 15]. In *Drosophila*, *Ofut1* (the *Pofut1* fly ortholog) mutants show severe Notch-like phenotypes [16]. If downregulation of *Ofut1* in signal-receiving S2 cells affects Notch ligands (Serrate or Delta) binding, its overexpression enhances Serrate-Notch binding and inhibits Delta-Notch binding. Interestingly, Ofut1 has a chaperone-like function in addition to its fucose transfer activity, thus facilitating the folding and proper membrane localization of Notch receptors [17]. Overexpression of *Ofut1*^*R245A*^ (encoding a protein without detectable fucosyltransferase activity) can partially affect phenotype under Notch pathway control, independently of fucose transfer [18], suggesting a possible disruption in Notch folding and/or incorrect membrane targeting. Increased POFUT1 levels can lead to a possible stress of the ER, as observed in mouse C2C12 myoblast cells [19]. So human POFUT1 could also play a dual role, acting not only as a glycosyltransferase but also as a molecular chaperone.

Mutations in the *POFUT1* gene are associated with two distinct pathologies. In Dowling-Degos Disease 2 (DDD2), they conduce to *POFUT1* haploinsufficiency, reducing Notch pathway activity, disrupting melanin synthesis, and leading to the hallmark reticulated hyperpigmentation characteristic of this autosomal-dominant genodermatosis [20]. A more recently inherited p.Ser162 homozygous missense variant in POFUT1 was characterized in a patient [21]. The mutation does not affect POFUT1 level but lowers its activity to less than 10% and disrupts Notch-dependent pathways essential for embryonic development. Severe phenotypes, including microcephaly, global developmental delay, and cardiovascular anomalies, were reported.

In numerous cancers, the expression of *POFUT1* is very largely upregulated, often leading to an increased quantity of the enzyme. Since the first published study in 2013 about oral cancer [22], many of them show that a stronger expression of *POFUT1* exists in breast cancer [23, 24], lung cancer [25], esophageal cancer [26, 27], pancreatic cancer [28], gastric cancer [29, 30], colorectal cancer [31–33], head and neck squamous cell carcinoma [34], hepatocellular carcinoma [35, 36], salivary gland carcinoma [37], and glioblastoma [38–40].

Although the role of POFUT1 in cancer progression has drawn significant interest due to its involvement in regulating key cellular processes, the precise mechanisms by which POFUT1 contributes to tumor progression remain incompletely understood. Several key questions persist. What molecular mechanisms drive *POFUT1* overexpression in different cancers? How does POFUT1 enhance tumor progression, affecting different oncogenic pathways? Can *POFUT1* inhibition serve as a viable therapeutic strategy? This review aims to (1) summarize the current knowledge on *POFUT1* dysregulation in various cancers; (2) explore its functional consequences on proliferation, migration, and apoptosis; and (3) decipher the known mechanisms underlying its dysregulation.

## *POFUT1* dysregulation in numerous cancers

*POFUT1* dysregulation, particularly its overexpression, is frequently seen across many cancer types. Although *POFUT1* consistently contributes to tumor malignancy, its specific impacts on tumor progression vary depending on the type of cancer (Table 1).

**Table 1:** Summary of research studies on *POFUT1* mRNA expression, POFUT1 protein levels, and their links to cancer progression across different cancer types

Type of cancer	Authors	Year of publication	Number of samples	Expression of *POFUT1*	Sample	Detection methods	Phenotype
Bladder	[41]	2021	105	Downregulated	FFPE	RT-qPCR, IHC	Poor survival outcomes, increased tumor progression
Breast	[23]	2014	394	Upregulated	Tissues	Microarray	Longer RFS and OS in node-negative breast cancer patients
	[24]	2017	314	Upregulated	FFPE	IHC	Poor prognosis, tumor aggressiveness
Colorectal	[32]	2018	35	Upregulated	Tissues	RT-qPCR, WB, IHC	Cancer progression (tumor growth, invasion and metastasis)
	[31]	2018	6	Upregulated	FPPE	RT-qPCR, WB, IHC	Cancer progression
	[42]	2019	14	Upregulated	Tissues	CNV-The AccuCopy assay, WB	Cancer progression and stemness in synergy with PLAGL2
	[43]	2020	78	Upregulated	Tissues	RNAseq, WGS, IHC	Progression from adenoma to carcinoma
	[44]	2024	30	Upregulated	FPPE	IHC	Cancer progression and stemness in NMAC
Esophageal	[26]	2020	10	Upregulated	Tissues	RT-qPCR	Cancer progression (tumor growth, invasion and metastasis)
	[27]	2023	386	Upregulated	Tissues, serum	nano-LC-Q-TOF-MS/MS, SERPA, ELISA, WB, IHC	Elevated POFUT1 autoantibodies promoting disease progression and precancerous lesions in HGIN
Gastric	[29]	2017	51	Upregulated	Tissues	IHC	Tumor progression, poor differentiation, reduced apoptosis
Glioblastoma	[39]	2021	12	Upregulated	Tissues	RT-qPCR, WB	Aggressive tumor behavior, poor prognosis, shorter overall survival
Head and neck	[34]	2023	55	Upregulated	Tissues	RT-qPCR, WB, IHC	Tumor progression, perineural invasion
Liver	[35]	2016	253	Upregulated	FFPE, tissues	RT-qPCR, WB, TMAs, IHC	Tumor aggressiveness
	[36]	2024	90	Upregulated	Tissues	IHC	Stabilized PD-L1, enhanced immune evasion
Lung	[25]	2018	156	Upregulated	Tissues, plasma	ddPCR	Tumor progression. Potential diagnostic biomarker in plasma
Oral	[22]	2013	128	Upregulated	Tissues	RT-qPCR, WB, IHC	Aggressive tumor progression
Pancreatic	[28]	2021	844	Upregulated	Tissues	IHC	Increased angiogenesis, tumor aggressiveness
Salivary gland	[37]	2023	74	Upregulated	Tissues	RT-qPCR, WB, IHC	Tumor proliferation, increased cell survival

*CNV* copy number variation, *ddPCR* droplet digital polymerase chain reaction, *ELISA* enzyme-linked immunosorbent assay, *FFPE* formalin-fixed, paraffin-embedded, *HGIN* high-grade intraepithelial neoplasia, *IHC* immunohistochemistry, *nano-LC-Q-TOF–MS/MS* nano liquid chromatography quadrupole time-of-flight tandem mass spectrometry, *NMAC* non-mucinous adenocarcinoma, *OS* overall survival, *PD-L1* programmed death-ligand 1, *PLAGL2* pleomorphic adenoma gene-like 2, *RFS* recurrence-free survival, *RNAseq* RNA sequencing, *RT-qPCR* reverse transcription quantitative polymerase chain reaction, *SERPA* serological proteome analysis, *TMAs* tissue microarrays, *WB* western blot, *WGS* whole genome sequencing

### Cancers with *POFUT1* overexpression

*POFUT1* is significantly overexpressed in hepatocellular carcinoma (HCC), with around 79% of HCC samples showing a marked increase in *POFUT1* mRNA expression. In addition, moderate to strong POFUT1 immunostaining intensities were observed in around 56% of HCC samples. This high *POFUT1* expression was associated with more aggressive tumor characteristics, including larger tumor size, poor cellular differentiation, and vascular invasion, contributing to poor survival and high recurrence rates [35]. A recent study demonstrates the role of POFUT1 in promoting HCC progression and the immunosuppressive tumor microenvironment, as higher POFUT1 protein quantity leads to enhanced production and improved stability of programmed death-ligand 1 (PD-L1) on cancer cells [36]. This mechanism facilitates tumor immune evasion, undermining the therapies using immune checkpoint blockades (ICB). These are designed to block PD-1 and PD-L1, thereby restoring T cell function and enhancing the immune response against tumors. The presence of high *POFUT1* expression results in lower response rates and poorer clinical outcomes in patients treated with anti-PD-L1 or anti-PD-1 immune checkpoint inhibitors.

In cancers like oral squamous cell carcinoma (OSCC), glioblastoma (GBM), and head and neck squamous cell carcinoma (HNSCC), overexpression of *POFUT1* has also been associated with highly invasive and aggressive tumor behavior [22, 34, 39]. For example, in HNSCC, it was linked to perineural invasion, a strong predictor of cancer recurrence and metastasis [34]. However, in salivary adenoid cystic carcinoma (SACC) where around 81% of SACC patient samples have a high *POFUT1* expression, it is associated with a higher T classification of tumor size, but not with lymph nodes, distant metastasis, or overall clinical staging [37].

POFUT1 is also associated with angiogenesis development in pancreatic ductal adenocarcinoma (PDAC) [28]. Its expression is significantly upregulated and strongly associated with increased micro-vessel density, contributing to heightened tumor vascularization. It drives a more aggressive cancer phenotype, emphasizing the critical role of POFUT1 in tumor progression through vascular mechanisms.

In breast cancer, especially in invasive ductal carcinoma, high POFUT1 levels detected by IHC are statistically associated with more aggressive tumor characteristics such as lymph node metastasis and high histological grade (tumor grade 3 versus grades 1 and 2) [24]. High expression of *POFUT1* mRNA was found to be associated with poor prognosis, including both shorter overall survival (OS) and recurrence-free survival (RFS) in breast cancer patients. However, contrasting findings from another study conducted on node-negative patients who did not receive systemic therapy showed that higher *POFUT1* expressions were associated with better outcomes, underscoring the context-dependent nature of *POFUT1* prognostic role, which may vary by molecular subtypes and treatment approaches [23].

In gastric cancer, IHC analysis revealed detectable POFUT1 levels in 64% of the samples, with significantly higher levels compared to adjacent normal tissues [29]. *POFUT1* overexpression is significantly associated with aggressive tumor features, including advanced T classifications, higher N classifications, and alteration of cellular differentiation. Despite these associations, no significant correlation between *POFUT1* expression and OS was observed, though a trend toward poorer prognosis in patients with higher expression warrants further investigation.

In colorectal cancer (CRC), *POFUT1* has been more extensively studied and is consistently found to be significantly overexpressed in CRC tissues with a 2.5-fold increase compared to adjacent normal ones, making it a contributor to CRC progression [31, 32]. Its involvement is especially notable in the transition from adenoma to carcinoma, with its overexpression often linked to high-risk adenomas that are more likely to develop into invasive cancer [43]. The differential expression of *POFUT1* is associated with tumor sites and pathologic stages of CRC [31]. Its overexpression is even more pronounced in non-mucinous adenocarcinomas (NMAC). Interestingly, there is an inverse relationship between the expressions of *POFUT1* and *MUC2*, a marker of mucinous adenocarcinoma (MAC), suggesting that *POFUT1* influences tumor differentiation and progression specifically in the NMAC subtype [44].

POFUT1 may also serve as an early biomarker in esophageal squamous cell carcinoma (ESCC) and non-small cell lung cancer (NSCLC), where *POFUT1* is overexpressed in tumor tissues and promotes cancer progression. In both cancer types, potential diagnostic biomarkers linked to POFUT1 were found in the patient bloodstream. In ESCC, POFUT1 autoantibodies have been detected in the serum at early stages of the pathology, providing a non-invasive diagnostic tool for early detection [27]. The receiver operating characteristic (ROC) curve based on the sensitivity and the specificity of this potential biomarker shows area under the curve (AUC) values ranging from 70.3 to 74.1% for POFUT1 autoantibodies, demonstrating a rather performant diagnostic accuracy, which can be improved by combining it with another biomarker. Similarly, in NSCLC, elevated plasma *POFUT1* mRNA levels strongly correlate with tumor progression, offering notable diagnostic performance, with high sensitivity (81.76%) and specificity (86.26%) [25]. This makes POFUT1 a valuable new non-invasive biomarker for both diagnosis and patient monitoring in esophageal and lung cancers.

### Cancers with *POFUT1* downregulation

Interestingly, in contrast to its usual oncogenic role, in muscle-invasive bladder cancer (MIBC), *POFUT1* is downregulated [41]. This reduced expression is associated with poorer survival, suggesting a tumor-suppressive role in this context. Patients with low *POFUT1* mRNA expression showed a 4.4-fold greater risk of a shortened lifespan regarding cancer-specific survival (CSS), compared to those with higher expression levels. Furthermore, OS and the disease-free survival (DFS) are impacted by a 2.6- and a 2.9-fold greater risk, respectively. The downregulation of the *POFUT1* gene associated with a reduced amount of the corresponding protein in MIBC can be linked to the inactivation of the Notch signaling pathway, a known tumor-suppressor mechanism in bladder cancer [41, 45]. In mice with induced gene knockout for *Psen1*/*Psen2* or *Rbpj* (key components of the Notch pathway) and treated by a carcinogen with high selectivity for the urothelium, the loss of Notch activation causes epithelial-mesenchymal transition.

**Fig. 2:**
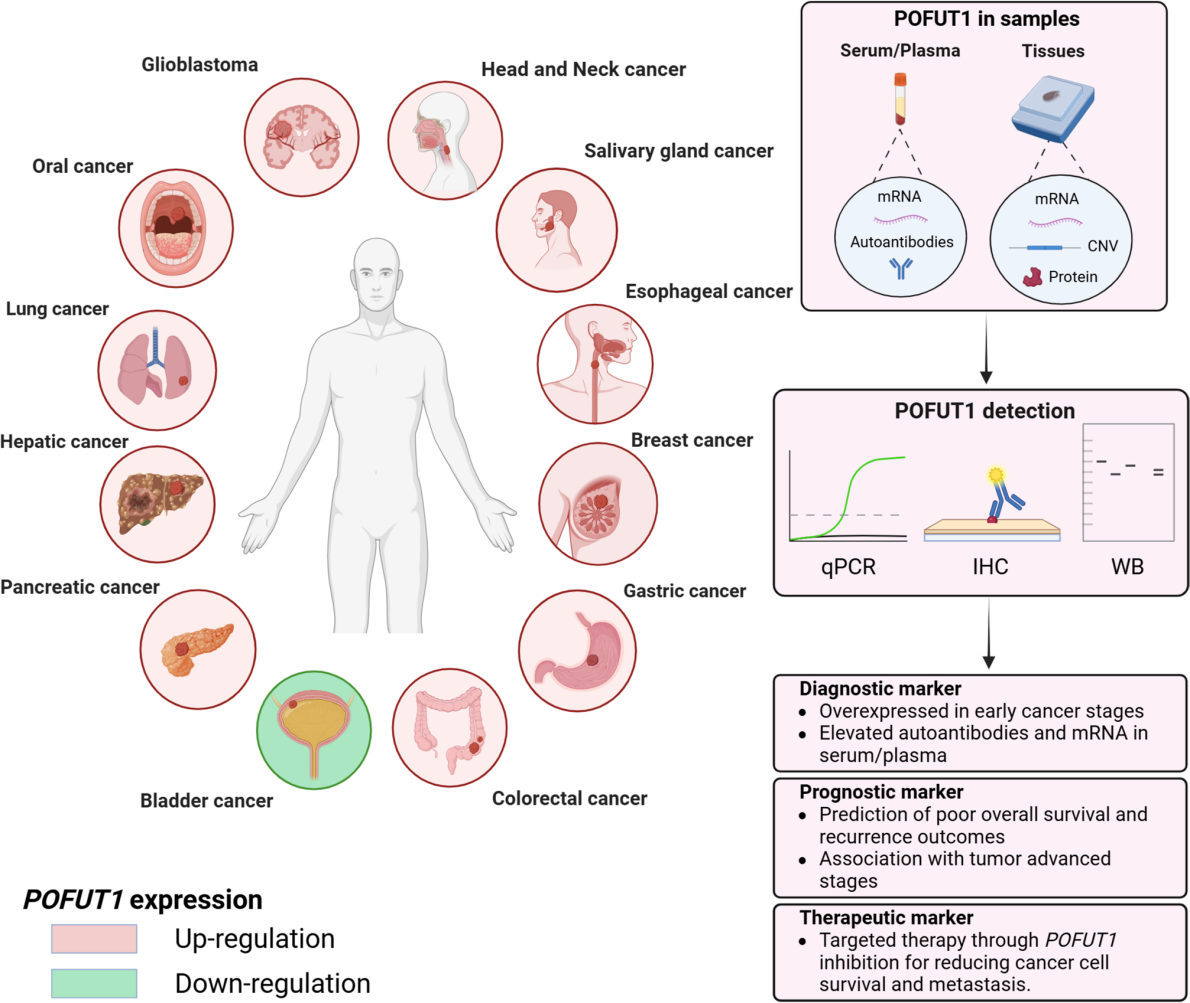
Cancer types with POFUT1 dysregulation. Its expression is predominantly upregulated (pink), except in bladder cancer, where it is downregulated (green). It is detectable in both serum/plasma and/or tumor tissues in various forms: mRNA, protein, autoantibodies, and copy number variations (CNVs). Quantitative PCR (qPCR), immunohistochemistry (IHC), and western blot (WB) are used to detect and quantify their presence. POFUT1 is emerging as a promising biomarker for precision oncology, offering new avenues for early detection, disease monitoring, and potentially targeted therapeutic strategies. Created by biorender.com

Therefore, POFUT1’s role is context-dependent, acting as an oncogene in most cancers, its overexpression tied to tumor aggressiveness, invasion, and poor survival, often through mechanisms like angiogenesis and immune evasion, but potentially serving as a tumor suppressor in select malignancies such as MIBC. Understanding these cancer-type-specific differences is crucial for developing accurate diagnostic tools and targeted therapeutic strategies (Fig. 2). Further investigations are needed to determine whether POFUT1 acts as a tumor suppressor in additional cancers. It could help clarify the molecular factors linked to POFUT1, dictating promotion or suppression of tumor growth, and assess whether a return to normal *POFUT1* expression can improve survival outcomes. Such considerations underscore the complexity of POFUT1’s role in cancer and highlight the need for cancer-specific therapeutic strategies.

## Role of *POFUT1* dysregulation in modulating signaling pathways in human cancer cells

The dysregulation of *POFUT1* has profound effects on multiple signaling pathways critical to cancer development.

### The Notch signaling pathway

The Notch signaling pathway plays a critical role in cell differentiation, proliferation, and apoptosis. It is highly conserved across animal species and becomes dysregulated in various cancers [46, 47]. In humans, the NOTCH receptors comprise between 29 and 36 EGF-LDs with 14 to 20 *O*-fucosylation consensus sites [7] that are mostly modified by *O*-fucosylation. In mice, only EGF-LDs 23, 24, and 32 NOTCH1 are not *O*-fucosylated [11]. If the initial *O*-fucose modification is catalyzed by POFUT1, this core fucose is often elongated by a β−1,3-N-acetylglucosaminyltransferase of the Fringe family (LFNG, MFNG, RFNG), resulting in the formation of the GlcNAc-β1,3-Fuc disaccharide. In mammals, this disaccharide is further extended by the sequential addition of one galactose and one sialic acid, producing the mature tetrasaccharide structure Sia-α2,3/6-Gal-β1,4-GlcNAc-β1,3-Fuc [48, 49]. The elongated structure is critical for ligand interactions necessary for canonical pathway activation [50]. Transmembrane ligands such as Jagged or Delta-like from adjacent cells bind to NOTCH receptors of the receiving cell, triggering receptor cleavage and the release of the Notch intracellular domain (NICD). The cleaved NICD then translocates to the nucleus where it interacts with transcription factors, such as RBP-Jk, to activate target genes like *HES1* and *HEY1*, which regulate cell proliferation and differentiation [51, 52]. Beyond its fundamental role in the elaboration of the *O*-fucosylglycan needed for modulation of Notch interaction with its ligands, *O-*fucosylation contributes to intra-molecular stabilization by interacting with nearby amino acids in the EGF-LD. This interaction enhances the structural integrity of NOTCH and accelerates its folding in the ER [6].

*POFUT1* dysregulation enhances Notch signaling in various cancers, where its overexpression leads to higher NICD levels and increased transcriptional activity of Notch target genes. This results in unchecked cell proliferation and reduced apoptosis, which contributes to tumor growth and progression [24, 32, 35]. Knockdown studies in colorectal, glioblastoma, and gastric cancer models have shown that reducing *POFUT1* expression significantly decreases cleaved NICD levels, leading to lower expressions of *HES1* and *HEY1* and reduced tumor cell proliferation and invasion [30, 32, 39]. In vivo studies, using subcutaneous xenograft models in nude mice, confirm the role of POFUT1 in tumor promotion through Notch signaling activation. For glioblastoma [39], *POFUT1* knockdown reduces tumor volume and weight linked to decreased Notch activation (lower NICD, HES1, and HEY1 levels). Similarly, for CRC [32], *POFUT1* silencing leads to smaller tumors in xenograft mice with reduced Notch activity, evidenced by diminished NICD, HES1, and c-Myc quantities. For gastric cancer [30], overexpressing *POFUT1* enhances both tumor growth and metastasis, with higher NICD levels and an additional synergistic effect with the Wnt pathway. These findings underscore POFUT1 as a key regulator of oncogenic Notch signaling, highlighting its potential as both a prognostic biomarker and a therapeutic target in cancers driven by aberrant Notch pathway activation.

### The Wnt/β-catenin signaling pathway

The Wnt/β-catenin signaling pathway is conserved in animals where it regulates fundamental biological processes such as embryonic development, tissue homeostasis, and cell differentiation [53]. The activation of this pathway occurs when Wnt proteins bind to transmembrane receptors called Frizzled, in conjunction with co-receptors like LRP (lipoprotein receptor-related protein) [54]. This interaction prevents the degradation of β-catenin, a pivotal protein in the pathway, allowing it to accumulate in the cytoplasm and subsequently translocate to the nucleus. The β-catenin functions there as a transcriptional co-activator with TCF/LEF transcription factors, initiating the expression of target genes involved in cell proliferation, migration, and survival [55]. Dysregulation of this pathway has been strongly linked to various diseases, particularly cancers. Aberrant Wnt signaling leads to the stabilization and the nuclear accumulation of β-catenin, which interacts with TCF/LEF to drive expression of the proto-oncogenes such as *CCND1* (encoding Cyclin D1) and *MYC*, fueling uncontrolled cell proliferation and tumor growth [56].

POFUT1, traditionally associated with the Notch signaling pathway, has been shown to enhance Wnt/β-catenin signaling, especially in colorectal cancer, glioblastoma, and gastric cancer. It promotes the formation of a parafibromin-NICD1-β-catenin complex, which stabilizes β-catenin and enhances the transcription of both Notch and Wnt target genes. This amplifies the oncogenic effects of both pathways [30]. The crosstalk between Notch and Wnt signaling creates a robust pro-tumorigenic environment where Notch activation suppresses Wnt antagonists, further increasing β-catenin activity [29, 30]. In gastric cancer, this interaction would promote cancer stemness, tumor growth, and metastasis, making treatment with single-pathway inhibitors less effective [30]. Targeting this synergistic relationship between Notch and Wnt pathways may provide a more comprehensive approach in combating aggressive cancers.

### The PI3K/AKT/mTOR signaling pathway

The PI3K/AKT/mTOR signaling pathway is a critical regulator of cellular processes such as growth, survival, metabolism, and proliferation. Activation begins with phosphoinositide 3-kinases (PI3Ks) producing PIP3 at the plasma membrane, which recruits and activates AKT, a serine/threonine kinase. The AKT phosphorylates downstream targets, including mTOR, a master regulator involved in protein synthesis, cell growth, and metabolic homeostasis [57, 58]. Dysregulation of this pathway is implicated in various diseases, notably in cancer where mutations in PI3K, loss of the tumor suppressor PTEN, or overactivation of AKT/mTOR drive tumor growth [59–61].

In trophoblast cells, POFUT1 has been studied for its role in regulating the PI3K/AKT/mTOR signaling pathway. Direct modulation of its expression revealed that *POFUT1* silencing inhibits the activation of signaling molecules (p-PDK, p-AKT308, and p-AKT468), leading to a decreased cell invasion. Conversely, the addition of leukemia inhibitory factor (LIF), a cytokine involved in the regulation of embryonic development and implantation, in the *POFUT1*-silenced cells increases POFUT1 amounts, restores PI3K/AKT/mTOR pathway activation, and enhances the invasive capacity of trophoblast cells [62]. Epiregulin, belonging to the EGF family and binding to some EGF receptors, increases *POFUT1* expression in trophoblast cells, which in turn catalyzes the *O*-fucosylation at Thr18 in the unique EGF-LD of uPA (urokinase-type plasminogen activator) [63]. This modification facilitates the interaction between uPA and its receptor uPAR [64], thereby contributing to PI3K/AKT/mTOR pathway activation. It is associated with the induction of epithelial mesenchymal transition (EMT) [63]. The link between POFUT1 and the PI3K/AKT/mTOR signaling pathway has not been really investigated in cancer. A study on glioblastoma demonstrates that the knockdown of *KDELC2* (encoding protein *O*-glucosyltransferase 3 targeting some EGF-LDs of NOTCH receptors) lowered *POFUT1* transcript amount as well as those of *NOTCH1-3* and *HES1*. It reduces p-PI3K, p-mTOR, and p-AKT quantities, implying that POFUT1 may indirectly contribute to the maintenance of the PI3K/AKT/mTOR pathway through regulation of the Notch one [65].

## Functional consequences of dysregulated *POFUT1* expression

**Fig. 3:**
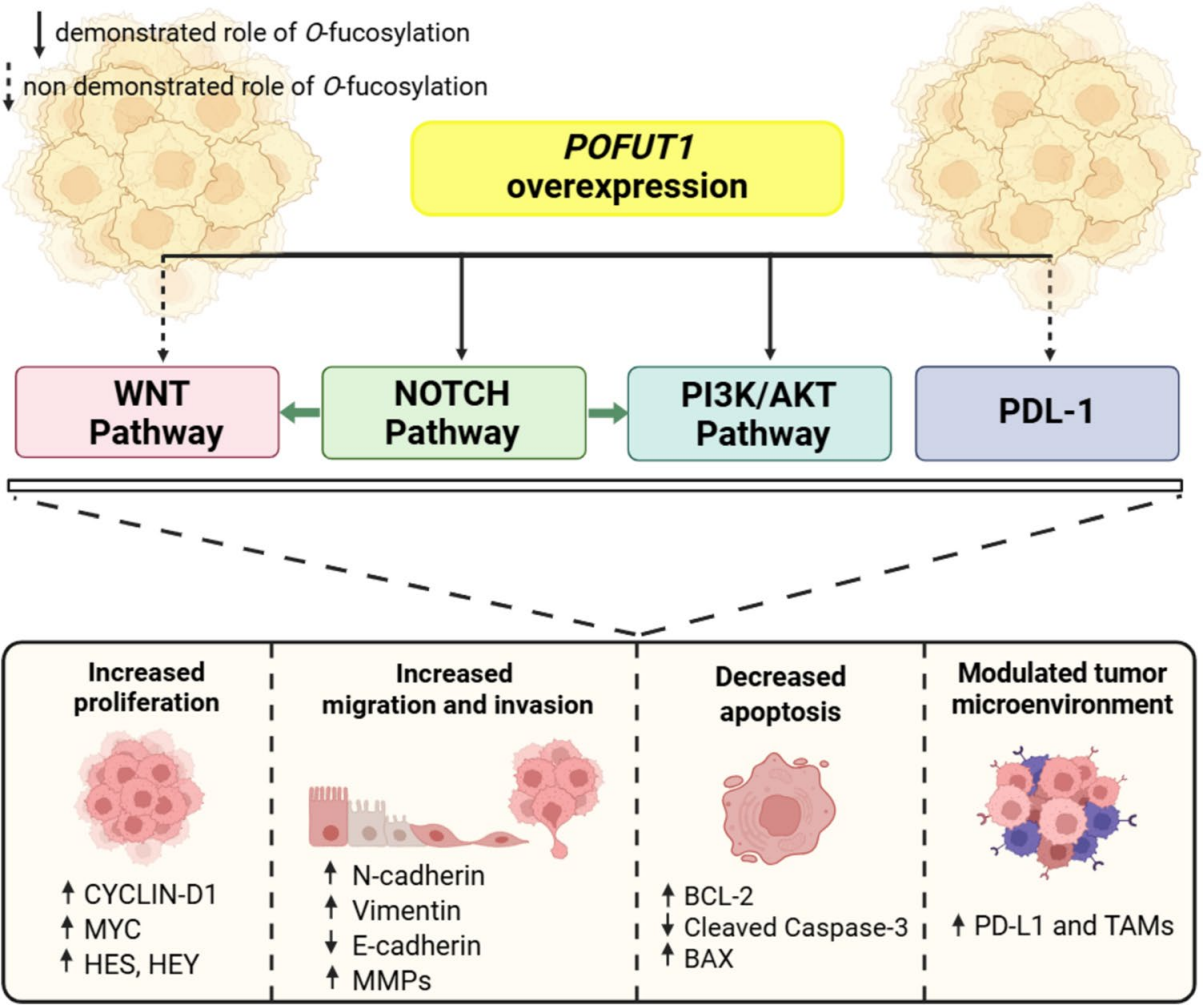
Overexpression of *POFUT1* activates key signaling pathways, including NOTCH, WNT, and PI3K/AKT and stabilizes PD-L1, which collectively promote cancer progression. POFUT1 dysregulation enhances cellular proliferation, facilitates migration and invasion, reduces apoptosis, and alters the tumor microenvironment to support immune evasion and tumor growth. The impact of the NOTCH pathway on others is represented by green arrows. Created by biorender.com

The cellular consequences of *POFUT1* deregulation are numerous, involving various signaling pathways, escape from the control of the immune system, and certainly other targets not yet discovered (Fig. 3).

### On cellular proliferation

Several studies demonstrate that in various cancers like glioblastoma, colorectal cancer, and hepatocellular carcinoma, overexpression of *POFUT1* promotes cancer cell proliferation [33, 35, 39]. This finding was confirmed by xenograft models, where tumors formed by *POFUT1* overexpressing cells grew faster and larger, with an average tumor volume of 2.79 ± 0.58 cm^3^ compared to 0.48 ± 0.22 cm^3^ for the control group [30]. In contrast, when *POFUT1* was silenced, a marked reduction in cell proliferation was observed, suggesting a strong link between *POFUT1* expression and tumor cell growth [32, 39]. Moreover, silencing *POFUT1* led to a clear cell cycle arrest, particularly at the G1 phase, indicating that POFUT1 is crucial for the transition to the S phase, where DNA replication occurs. This supports the conclusion that POFUT1 actively drives cancer cell proliferation [32, 33].

### On migration and invasion

*POFUT1* has been shown to significantly enhance the migration and invasion abilities of cancer cells. In gastric cancer, transwell migration and invasion assays were used to compare the behavior of *POFUT1* overexpressing cells with control ones. Cells with higher *POFUT1* expression exhibited a substantial increase in migration and invasion capabilities. This was further supported by IHC staining, which revealed increased levels of matrix metalloproteinases (MMPs), particularly MMP-9, in *POFUT1* overexpressing cells, facilitating the breakdown of the extracellular matrix, a critical step in invasion [30]. In colorectal cancer, it has been demonstrated that knocking down *POFUT1* in CRC cells significantly reduced their migratory and invasive potentials [32, 33]. For example, in transwell migration assays, the number of migratory cells decreased from 639 ± 48 per field in control SW620 colon cancer cell line to 331 ± 46 per field in *POFUT1*-silenced SW620 control *vs* *POFUT1*-silenced cells, and from 524 ± 48 per field to 281 ± 36 per field in HCT116 colon cancer cell lines [32]. Furthermore, in vivo metastasis experiments in mice, where colon cancer cell lines with high and low *POFUT1* expression were injected, demonstrated that cells with high *POFUT1* expression promoted metastatic nodules in the liver, confirming its role in enhancing invasion [32]. In glioblastoma (GBM), knocking down *POFUT1* in GBM cell lines induces a significant reduction in cell migration and invasion in both wound healing and transwell invasion assays. These results were also confirmed in vivo using mouse models, where the number of metastases and tumor invasiveness were significantly reduced upon *POFUT1* knockdown [39].

### On epithelial-mesenchymal transition (EMT)

*POFUT1* dysregulation significantly influences the epithelial-mesenchymal transition (EMT), facilitating cell migration, invasion, and metastasis. In human trophoblast cells, POFUT1 has been shown to activate the PI3K/AKT/mTOR signaling pathway, which in turn drives EMT. This activation is mediated through *O*-fucosylation-dependent enhancement of uPA/uPAR signaling [63]. It promotes EMT by increasing mesenchymal markers like N-cadherin and vimentin, reducing epithelial markers such as E-cadherin, thereby enhancing trophoblast migration and invasion, which are critical for embryo implantation. Higher levels of *POFUT1* and epiregulin are correlated with successful implantation, while their reduction is associated with impaired EMT and abortion [63]. Similarly, in head and neck squamous cell carcinoma, *POFUT1* overexpression is associated with perineural invasion (PNI), where increased N-cadherin and vimentin and decreased E-cadherin levels support the EMT-driven invasive phenotype [34].

### On apoptosis

POFUT1 also plays a crucial role in inhibiting apoptosis, thus promoting tumor cell survival. In gastric cancer, Dong et al. [30] demonstrated through flow cytometry-based apoptosis assays that *POFUT1* knockdown in cancer cells significantly increased apoptosis. The quantity of pro-apoptotic markers, such as cleaved caspase-3 (CASP3) and Bcl2-associated X protein (BAX), is increased in *POFUT1*-silenced cancer cells, while anti-apoptotic proteins like BCL2 decreased. These results were validated in subcutaneous mouse xenograft models, where *POFUT1*-silenced tumors showed increased apoptosis and were significantly smaller compared to control tumors [30, 32, 33]. Similarly, observations were made in colorectal cancer, where *POFUT1* knockdown led to a significantly higher proportion of apoptotic cells, with apoptosis rates increasing from 10.56% ± 1.33% in control SW620 cells to 17.23% ± 2.57% in *POFUT1*-silenced SW620 cells, and from 14.54% ± 1.78% to 25.91% ± 0.51% in control and *POFUT1*-silenced HCT116 cells, respectively [32]. It was confirmed by mouse xenografts, where tumors with reduced *POFUT1* expression displayed DNA fragmentation, a hallmark of apoptosis, as measured by TUNEL assays.

These experimental findings highlight the significant impact of POFUT1 on cancer cell proliferation, migration, invasion, and apoptosis. Consistent in vitro and in vivo results show that *POFUT1* overexpression promotes tumor growth and metastasis, while its knockdown reduces cell proliferation and migration and increases apoptosis. It supports the notion that POFUT1 is a cancer driver participant, acting on its progression and on cancerous cellular survival, synergistically with other impaired oncogenes and/or tumor suppressor genes.

### On tumor microenvironment

Recent studies have highlighted the role of POFUT1 in modulating the tumor microenvironment, particularly through its effect on immune evasion. POFUT1 has been shown to stabilize PD-L1 on the surface of cancer cells by preventing its ubiquitination and subsequent degradation [36]. PD-L1 is a molecule that interacts with PD-1 on T cells, inhibiting their activation and reducing their cytotoxic response. By increasing PD-L1 levels, POFUT1 suppresses the infiltration and activity of anti-tumor immune cells, such as CD8 + T cells, thereby fostering an immunosuppressive microenvironment. Notably, this regulatory effect is independent of the fucose transfer activity of POFUT1. Although POFUT1 typically modifies proteins containing EGF-LD, PD-L1 lacks this structural motif. Instead, POFUT1 interacts with tripartite motif containing 21 (TRIM21), an E3 ubiquitin ligase. This interaction prevents PD-L1 ubiquitination and proteasomal degradation, allowing its accumulation at the cell surface and the maintenance of its immunosuppressive function. In gliomas, particularly low-grade ones (LGG), high *POFUT1* expression correlates with poor patient prognosis and increased infiltration of immune-suppressive cells, notably M2-like tumor-associated macrophages (TAMs), regulatory T cells (Tregs), and resting memory CD4 + T cells [40]. It fosters an immune microenvironment favoring TAMs, which are linked to tumor progression. The ability of POFUT1 to shape a part of the immune microenvironment underscores the need for future interest in this marker as a therapeutic target, particularly in immunotherapy resistance.

## Mechanisms of *POFUT1* dysregulation

### Copy number variation

**Fig. 4:**
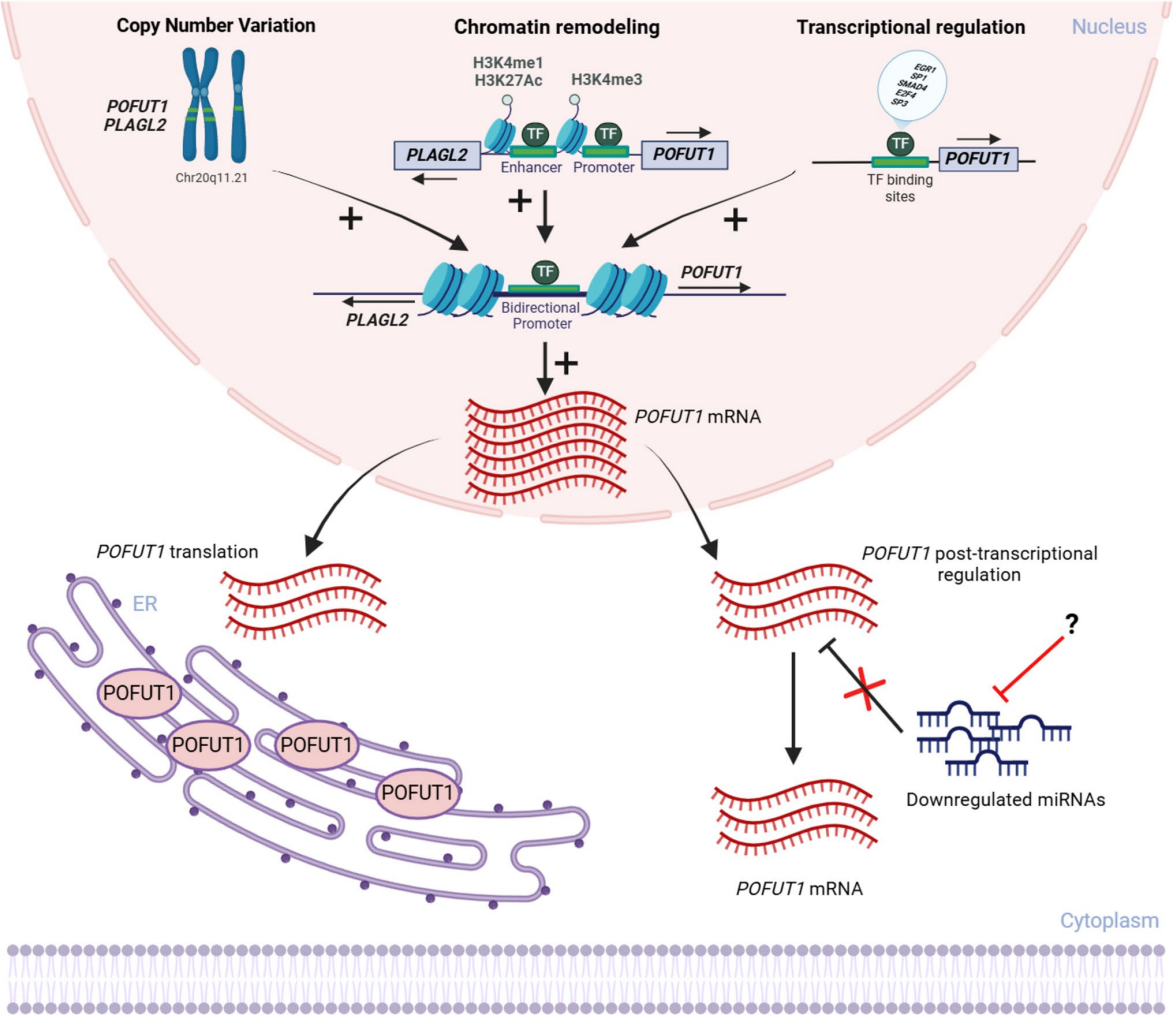
Mechanisms underlying *POFUT1* dysregulation in cancer, highlighting both nuclear and cytoplasmic processes. In the nucleus, *POFUT1* overexpression is driven by copy number variations of region 20q11.21, chromatin remodeling, and transcriptional regulation through transcriptional factors (TF). The bidirectional promoter shared with *PLAGL2* allows their coordinated transcription. In the cytoplasm, the increased amount of *POFUT1* mRNAs could be split into two pools, one translated and promoting enzyme accumulation in the endoplasmic reticulum (ER) and the other, escaping the post-transcriptional regulation by miRNAs targeting *POFUT1*. The cause of the miRNA decrease is currently unknown as well as the exact role of *POFUT1* mRNA, which could serve as an RNA sponge. The mechanisms of *POFUT1* overexpression would act separately or in combination, then affecting cancer progression. Created by biorender.com

The mechanisms underlying the deregulation of *POFUT1* remain incompletely understood. However, recent studies suggest that this deregulation is primarily driven by copy number variations (CNVs), which are significant genomic alterations contributing to cancer development [66, 67]. CNVs are a common feature in acute myeloid leukemia and colorectal cancer, with frequent gains, like those seen on chromosome 20q [68, 69]. Losses on 8p were also found in CRC, which combined with CNV of chromosome 20q, amplified tumor progression and conduced to a poor prognosis. A key consequence of CNV-driven gene amplification in CRC is the overexpression of *POFUT1*, located in the 20q11.21 region (Fig. 4). This region is frequently amplified in 7 to 9% of colorectal cancers [70]. The resulting *POFUT1* amplification leads, among others, to elevated levels of *POFUT1* mRNA and protein, due to a gene dosage effect. These changes fuel abnormal cell growth and proliferation, accelerating oncogenic transformation and tumor progression [42]. Interestingly, a study highlighted that CRC cell lines with *POFUT1* amplification exhibit resistance to certain chemotherapy drugs, especially microtubule poisons and some mitotic inhibitors but may be more sensitive to Notch pathway inhibitors [71]. At the position q11.21 of chromosome 20, *POFUT1* is adjacent to *PLAGL2* (pleomorphic adenoma gene like-2), a transcription factor known for its critical role in promoting EMT by interacting with β-catenin in the context of the Wnt signaling pathway. *PLAGL2* enhances the expression and nuclear translocation of β-catenin by decreasing its phosphorylation. When activated, β-catenin forms a complex with TCF/LEF and binds to the promoter of the gene encoding the transcription factor ZEB1 (zinc finger E-box binding homeobox 1), enhancing its expression. Increased levels of ZEB1 then suppress E-cadherin expression, while simultaneously promoting the expression of N-cadherin and vimentin, which are key markers of the mesenchymal phenotype in EMT [72]. PLAGL2 is also identified as a prognostic marker, correlating with advanced cancer stages and poor outcomes [73, 74]. *POFUT1* and *PLAGL2* share a short evolutionary conserved (around 300 bp) bidirectional promoter [42, 75, 76]. These two genes, positioned on opposite strands of DNA, share this promoter in a head-to-head configuration, meaning their expression is coordinated, ensuring both genes are simultaneously expressed. In cancers with CNVs of the region, their coordinated upregulation leads to a synergistic effect, driving Notch and Wnt signaling pathways that promote tumor development, progression, and aggressiveness.

### Epigenetic control of gene expression

While CNVs are a major driver of *POFUT1* overexpression in acute myeloid leukemia [68] and colorectal cancer [69] as mentioned above, other mechanisms could also contribute, such as epigenetic control of transcription. Epigenetic changes, such as DNA hypomethylation or histone modifications, can explain gene overexpression [77]. Bioinformatics analysis of the bidirectional promoter region of *POFUT1*/*PLAGL2*, enriched with histone marks associated with active transcription (H3K4me3, H3K4me1, and H3K27Ac), suggests chromatin remodeling and an open chromatin state conducive to transcription (Fig. 4) [78]. This, along with the presence of a CpG island in the intergenic region between *POFUT1* and *PLAGL2*, indicates that DNA methylation and histone modifications may influence *POFUT1* dysregulation in cancer.

### Transcriptional and post-transcriptional regulations

On the transcriptional front, *POFUT1* with *PLAGL2* share, in their bidirectional promoter, over 160 predicted sites for transcription factors (TF) [78]. They include EGR1, SP1, SMAD4, E2F4, and SP3, which are predicted to contribute to the coordinated expression of both genes (Fig. 4). Alterations of TF presence in the promoter region may conduce to activate or repress *POFUT1* expression. In this context, a study demonstrated that Caveolin-1 (CAV1) upregulates *POFUT1* expression in hepatocellular carcinoma by activating the MAPK signaling pathway. This activation leads to the phosphorylation of transcription factors such as CREB, SP1, HNF4A, and MYC, which subsequently bind to the *POFUT1* promoter and enhance its transcription [79].

*POFUT1* overexpression could also result from a defective post-transcriptional regulatory mechanism, such as the one operated by microRNAs (miRNAs) (Fig. 4). *POFUT1* is identified as a conserved target of the miR-34 family across species, including mice, rats, chimpanzees, and humans [80]. Reduction of miR-34a may contribute to the overexpression of *POFUT1*. Notably, miR-34a-5p has been found to be downregulated in various cancers, including pancreatic [81], glioblastoma [82], and CRC [83], where *POFUT1* is overexpressed. In addition to *POFUT1*, miR-34a also targets *NOTCH1*, underscoring its central role in regulating oncogenic processes through modulation of the Notch signaling pathway [84, 85]. *POFUT1* and *PLAGL2* transcripts share 52 common miRNAs predicted through databases like TarBase (https://dianalab.e-ce.uth.gr/tarbasev9) and ENCORI/starBase (https://rnasysu.com/encori/) to directly target the 3′untranslated region (3′UTR) of both *POFUT1* and *PLAGL2*, which could repress their expressions in colorectal cancer [78].

Recently, a form of circular RNA derived from the *POFUT1* gene and restricted to exons 3 and 4 has been identified as a key driver in the progression, metastasis, and chemoresistance of CRC [86]. This circularization occurs during the transcription of *POFUT1* pre-mRNA and produces a stable RNA molecule that lacks free 5′ and 3′ ends. In normal cells, circRNAs are typically expressed at low levels and play regulatory roles in transcription, alternative splicing, and cell cycle control. In CRC cells, circ*POFUT1* is overexpressed and functions as a miRNA sponge, specifically sequestering miR-653-5p, normally targeting the 3′UTR of *E2F7*. This competition increases the quantity of the transcription factor E2F7, which subsequently upregulates downstream targets like WD repeat-containing protein 66 (WDR66) linked to EMT, promoting CRC metastasis and chemoresistance [86].

All these mechanisms could promote, independently or collectively, the overexpression of *POFUT1*, which in turn activates key signaling pathways, crucial for maintaining cancer cell growth, survival, and metastatic potential. However, only a few scientific studies were devoted to their characterization. Further experiments are needed to delineate the specific transcriptional and post-transcriptional regulatory mechanisms involved.

## Conclusion and future perspectives

Over the past decade, mounting evidence has revealed the pivotal role of POFUT1 in tumorigenesis processes. Increasing data demonstrate that POFUT1 plays a complex, context-dependent role in cancer biology, significantly impacting tumor progression, migration, invasion, immune evasion, and apoptosis across various cancer types. Frequently upregulated in cancers like colorectal, breast, and hepatocellular carcinoma, POFUT1 promotes aggressive phenotypes through its involvement in Notch, Wnt/β-catenin, and PI3K/AKT/mTOR signaling pathways. However, in certain contexts such as muscle-invasive bladder cancer, *POFUT1* downregulation suggests a potential tumor-suppressive function, underscoring its dual behavior as an oncogene and, very selectively, as a tumor suppressor gene. This nuanced behavior highlights the potential role of POFUT1 as both a biomarker and a therapeutic target, given its association with immune evasion mechanisms, particularly through PD-L1 stabilization, making it an intriguing candidate for enhancing immunotherapy efficacy in tumors with high *POFUT1* expression.

Targeting POFUT1 may offer new avenues of therapeutic strategies for cancer treatment. Inhibiting *POFUT1* expression or enzymatic activity could suppress tumor growth. Given that *POFUT1* expression could be regulated by DNA methylation and/or histone modifications, the use of epigenetic modulators such as DNA methyltransferases or histone deacetylase inhibitors could restore normal *POFUT1* expression levels, particularly in cancers where it is aberrantly overexpressed. The restoration of tumor-suppressive miRNAs that negatively regulate *POFUT1* expression could also help to limit its oncogenic effects. The development of small-molecule inhibitors targeting POFUT1 enzymatic activity could disrupt Notch and Wnt pathway hyperactivation, limiting tumor growth and metastasis. Understanding pathway crosstalk, especially among Notch, Wnt/β-catenin, and PI3K/AKT/mTOR, could uncover further therapeutic targets, while exploring drug development strategies that consider POFUT1’s differential role across cancers could improve treatment precision. However, drug selectivity and toxicity must be carefully evaluated given POFUT1’s role in normal physiological functions. Despite these promising perspectives, challenges remain in clarifying the underlying molecular mechanisms acting on *POFUT1* dysregulation, such as its copy number variations, epigenetic modifications at the DNA level, and miRNA interactions.

POFUT1 could also be used as a predictive biomarker. Found in the plasma of patients suffering from lung and esophageal carcinoma, circulating *POFUT1* could be used, in combination with others, as a plasma-based biomarker for noninvasive diagnostics. It could also serve as a prognostic biomarker, given the correlation between *POFUT1* expression levels and aggressive tumor behavior. It will be a predictive biomarker for patient stratification by identifying high-risk individuals who may benefit from targeted Notch/Wnt inhibitors or immune checkpoint blockade therapies.

In conclusion, POFUT1 has emerged as a crucial oncogenic player in multiple cancers, modulating key signaling pathways that drive tumorigenesis, metastasis, and immune evasion. Its regulation by copy number variations, epigenetic modifications, and post-transcriptional controls highlights its complexity and its potential for targeted interventions. While significant progress has been made in understanding POFUT1’s implication, further studies are required to translate this knowledge into effective clinical tools. By combining insights from genomics, epigenetics, and immuno-oncology, the development of POFUT1-targeted therapies could open new avenues for precision cancer treatment, offering hope for better patient outcomes in aggressive malignancies.

## Data Availability

Data sharing is not applicable to this article, as no datasets were generated or analyzed during the current study.
